# The First-Night Effect on the Instability of Stage N2: Evidence from the Activity of the Central and Autonomic Nervous Systems

**DOI:** 10.3390/brainsci13040667

**Published:** 2023-04-16

**Authors:** Ning Ma, Qian Ning, Mingzhu Li, Chao Hao

**Affiliations:** 1 Philosophy and Social Science Laboratory of Reading and Development in Children and Adolescents (South China Normal University), Ministry of Education, Guangzhou 510631, China; 2 Center for Sleep Research, Center for Studies of Psychological Application, Guangdong Key Laboratory of Mental Health & Cognitive Science, School of Psychology, South China Normal University, Guangzhou 510631, China

**Keywords:** first-night effect, EEG activation, heart rate variability

## Abstract

A series of studies have suggested that stage N2 is vulnerable and strongly affected by the first-night effect (FNE). However, the neurophysiological mechanism underlying the vulnerability of stage N2 of the FNE has not been well examined. A total of 17 healthy adults (11 women and 6 men, mean age: 21.59 ± 2.12) underwent two nights of polysomnogram recordings in the sleep laboratory. We analyzed sleep structure and central and autonomic nervous system activity during stage N2 and applied the electroencephalographic (EEG) activation index (beta/delta power ratio) and heart rate variability to reflect changes in central and autonomic nervous system activity caused by the FNE. Correlation analyses were performed between EEG activation and heart rate variability. The results showed that EEG activation and high-frequency heart rate variability increased on the adaptation night (Night 1). Importantly, EEG activation was significantly associated with the percentage of stage N1, and the correlation between EEG activation and high-frequency heart rate variability decreased due to the FNE. These findings indicate that the FNE affects the instability of stage N2 by increasing central nervous system activity and uncoupling the activity between the central and autonomic nervous systems.

## 1. Introduction

The first-night effect (FNE) is when sleep quality declines in an unfamiliar environment [1]. Several factors are supposed to contribute to the FNE, such as an unfamiliar sleep environment, discomfort provoked by electrodes, and monitoring [2,3]. Besides the influence on sleep structure, such as lower sleep efficiency, longer sleep latency, more wakefulness after sleep onset (WASO), and increased percentage of sleep stage N1 [3,4,5,6], the FNE also impacts the activity of the central nervous system (CNS) and the autonomic nervous system (ANS).

Recent studies indicated that EEG activity during NREM sleep was strongly affected by the FNE [5,7,8]. Shirota and colleagues found that the continuity time for stage N2 was significantly shorter on the adaptation night (Night 1) than on the experimental night (Night 2) and that there was no difference in stage N3. They also found that the transition probability from stage N2 to stage N1 was increased, and the transition probability from stage N2 to stage N3 was decreased on Night 1 compared to Night 2, while there was no difference in the transition probability from stage N3 to other stages between the two nights [7]. In addition, the FNE is a transient insomnia model [4], and quantification of sleep-stage dynamics revealed a particular vulnerability of stage N2 in insomnia, with increased transition probability from stage N2 to stage N1 and WASO [9]. These findings indicated that stage N2 was vulnerable and strongly affected by the FNE. Therefore, we focused on stage N2 to further explore the effect of the FNE in the current study.

The wake–sleep state control network commonly modulates the CNS and ANS, mainly located in the hypothalamus and brainstem [10]. Furthermore, the CNS and ANS are closely associated during sleep, which is reflected in the reciprocal oscillation between cortical and cardiac activity [10,11]. A series of studies elucidated that beta and delta power was inversely related to the high-frequency (HF) component of heart rate variability (HRV) during NREM sleep [12,13,14]. The HRV is one of the principal ANS activity indexes. High-frequency band (HF-HRV, 0.15–0.4 Hz) activity reflects parasympathetic activity and correlates with NREM sleep depth [15]. The contradictory relationship of delta and beta oscillation with HRV during sleep suggested the EEG activation index, which is one integrated index of EEG activity, calculated by the ratio of beta to delta power. This index might be more suitable to quantify the coupling of CNS and ANS activity during sleep. Furthermore, the EEG activation index has proved to be a reliable index to evaluate arousal level [16,17]. As this index increases, the brain moves toward a more wake-like pattern [18].

Since the coupling of the CNS and ANS is closely related to stable sleep states [10], exploring the FNE in relation to the coupling of CNS and ANS activity might help understand the underlying mechanism of the vulnerable stage N2 of the FNE. A recent study investigated the change in CNS and ANS activity during the first night. The results showed that the FNE increased the high beta power and decreased the index of HRV (including the HF-HRV and R-to-R wave intervals) during the first NREM sleep cycle [7], which indicated that the change in EEG activity might be associated with the variation in HRV caused by the FNE. However, the effect of the FNE on the coupling of CNS and ANS activity has not been well examined.

Therefore, the primary aim of the current study was to examine the FNE during stage N2 through the CNS and ANS activity. We used the EEG activation index and HRV to reflect the CNS and ANS activity changes caused by the FNE. Furthermore, by examining the correlation of EEG activation with HRV and whole-night sleep structure, respectively, we explored the neurophysiological mechanisms underlying the vulnerability of stage N2 of the FNE. Based on the previous studies [5,7,19], we hypothesized that EEG activation increased on Night 1 but not on Night 2 and that the increased EEG activation was associated with the changes in sleep structure and HRV.

## 2. Methods

### 2.1. Participants

Seventeen healthy college students (11 women and 6 men, mean age: 21.59 ± 2.12, BMI: 19.73 ± 1.58 kg/m^2^) participated in the study. They were assessed by a structural screening questionnaire. The screening criteria of the participants included: (1) right-handed; (2) no shift work or transmeridian travel within one month preceding the experiment; (3) no history of sleep-related disorders or mental or psychiatric disorders or disease; (4) no smoking or drinking habits, caffeine dependence, or history of substance abuse or drug use; (5) no acute or chronic disease; (6) normal sleep–wake habits, such as sleep 6.5–9 h per night, going to bed no later than 12:30 at night and getting up between 6:30 am and 9:00 am.

We used the Morningness and Eveningness Questionnaire (MEQ) [20] to measure individuals’ chronotypes. Extreme morning-type and evening-type individuals were excluded, and the participant’s scores in the present study were between 43 and 69. The Pittsburgh Sleep Quality Index Questionnaire (PSQI) [21] was used to assess sleep quality and disturbances over one month, and the scores of the participants in our study were ≤5. All study procedures were approved by the Ethical Committee of the South China Normal University and were conducted in accordance with the Declaration of Helsinki. All participants signed informed consent before participating in the study.

### 2.2. Procedures

One week before the experiment, participants were instructed to maintain their regular sleep–wake habits, including 6.5–9 h daily sleep hours, falling asleep before 12:30 pm, and waking between 6:30 am and 9:00 am. Sleep–wake patterns were recorded using sleep diaries, and we confirmed that regular sleep–wake habits were maintained (see Table 1). The participants were not allowed to perform excessive exercise or consume any beverages or foods containing caffeine, such as tea or coffee, 48 h prior to the experiment.

After a week of sleep monitoring, participants came to the sleep laboratory for two nights of polysomnogram (PSG) recordings. Each night, participants arrived in the laboratory at about 8:00 pm, then the electrodes for PSG recording were attached. The participants stayed and slept in the sleep laboratory for the whole night. On the following day, they woke up between 7:00 and 7:30 am.

### 2.3. Polysomnography Recordings

Polysomnography was performed using the Compumedics Grael system (Compumedics, Abbotsford, Victoria, Australia). It comprised ten electroencephalogram (EEG, sampling rate: 512 Hz) electrodes placed according to the international standard 10/20 system (O1, O2, C3, C4, F3, F4, M1 and M2; with Fpz as the reference and Fp1 as the ground), two electrooculogram (EOG) electrodes, three submental electromyogram (EMG) electrodes, and two electrocardiogram (ECG, sampling rate: 1024 Hz) electrodes. Electrode impedance was kept below 5 kΩ. Sleep scoring was performed according to the American Academy of Sleep Medicine (AASM) criteria [22] within each 30 s epoch. In order to ensure that participants did not have any sleep-related disorders, we excluded individuals whose sleep efficiency was less than 85% on Night 2. As a result, all participants’ sleep efficiencies were greater than 85% on Night 2.

### 2.4. Quantitative Analysis of EEG Signals

The whole-night sleep EEG data were processed in MATLAB R2016a (The MathWorks Inc., Natick, MA). Continuous EEG signals were referenced offline to the left or right mastoid (M1 or M2). All signals were filtered using a 0.5 Hz high-pass filter, a 40 Hz low-pass filter, and a 50 Hz notch filter. Then, sleep recordings were divided into 6 s epochs, and artifacts in the 6 s epochs were visually removed. Portions in which the absolute amplitude of the EEG signal exceeded 150μV were marked as artifacts. If more than 25% of artifacts were removed for one participant, this participant would be excluded from the analysis. Finally, only one participant was excluded from the spectral power analysis. Whole-night spectral power averages were obtained across all artifact-free epochs of stage N2.

Fast Fourier transformation (FFT) was used in MATLAB R2016a to calculate the spectral power density, and the truncation error was reduced by applying a Hanning window (50% overlap). The obtained power spectral data were divided into two bands (delta (0.5–4 Hz) and beta (13–30 Hz)). In this study, we only evaluated the beta/delta power ratio as an integrated EEG index of activation.

### 2.5. Quantitative Analysis of ECG Signals

Heart rate was taken from the PSG recording, representing autonomic nervous system activity. In a first step, we used MATLAB R2016a to compute the values for heart cycle duration from the raw ECGs, defined as the RR intervals, according to Perakakis [23]. All raw ECG signals were filtered using a 3 Hz high-pass filter and a 20 Hz low-pass filter. Then, after examining the ECG data and removing the artifacts, we analyzed the HRVs of the R-waves series across the whole-night stage N2 period using Kubios HRV Analysis Software 3.5 (MATLAB), according to Task Force guidelines [24]. We used fast Fourier transformation to quantify the absolute spectral power at high frequency (HF, 0.15–0.40 Hz), low frequency (LF, 0.04–0.15 Hz), and very low frequency (VLF, 0–0.04 Hz). These parameters were measured in square milliseconds [25]. The electrocardiogram electrodes fell off two participants on Night 2 and one on Night 1. Therefore, only 14 participants were included in the heart rate analysis.

### 2.6. Statistical Analysis

Paired *t*-tests (two-tailed) were used to compare the sleep structures, EEG activation indexes, and ECG spectral powers between Night 1 and Night 2. Furthermore, we computed Pearson correlations to test the correlation between sleep structure and EEG activation index for each night and the correlation between ECG spectral power and EEG activation index for each night. For post hoc analysis, the Benjamini–Hochberg procedure [26] was applied for correlation analysis, and corrected *p*-values were reported. As for effect size, we used Cohen’s *d* to evaluate the *t*-tests, which indicates the magnitude of the difference between two groups. It is interpreted as a large effect size when Cohen’s *d* ≥ 0.8 and as a medium effect size when *d* ≥ 0.5 [27]. Statistical significance was set at *p* < 0.05.

In the process of artifact deletion, the epoch deleted by one subject in stage N2 was greater than 25%. Therefore, 16 participants entered the final EEG spectral analysis. In addition, the electrocardio-electrodes fell off two participants on Night 2 and one on Night 1. As a result, we analyzed sleep structure for 17 participants, EEGs for 16 participants, and ECGs for 14 participants.

## 3. Results

### 3.1. The Whole-Night Sleep Structure

The sleep structures for Night 1 and Night 2 are presented in Table 2. The total sleep time was marginally shorter on Night 1 than on Night 2 (*p =* 0.054). A higher percentage of stage N1 (*p* < 0.001), longer sleep latency (*p* = 0.003), and more WASO (*p* < 0.001) were shown on Night 1 than Night 2. As a result, the sleep efficiency decreased on Night 1 compared to Night 2 (*p* < 0.001).

### 3.2. EEG Activation Index in Stage N2

In this study, EEG activation was calculated using the formula: [beta/delta]×100 (beta and delta presented absolute power of EEG), which was a reliable index for evaluating arousal level. The EEG activation results are shown in Table 3. In addition, paired *t*-tests compared the activation indexes in stage N2 for six scalp locations. The activation index was higher on Night 1 than on Night 2, and significant results appeared at the C4 and O2 electrodes (*p* < 0.05).

### 3.3. HRV Parameters in Stage N2

The differences in HRV parameters between Night 1 and Night 2 are shown in Table 4. Only HF amplitude was significantly higher on Night 1 than on Night 2 (*p* < 0.01). There were no significant differences between the two nights in other parameters of HRV.

### 3.4. Correlation Analysis of EEG Activation and Sleep Structure and HRV

**Figure 1:**
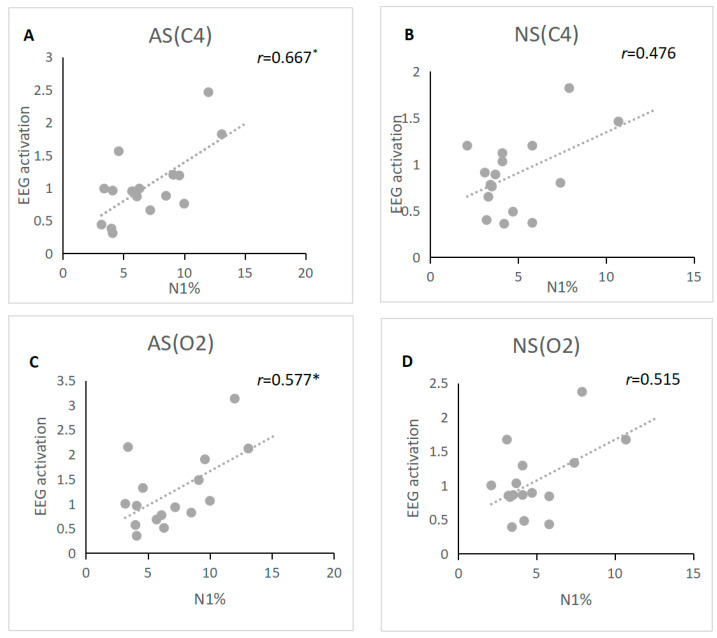
Association between the percentage of stage N1 and EEG activation during stage N2. Note: The relationship between the percentage of stage N1 and stage N2 EEG activation at the C4 electrode on Night 1 (shown in (**A**)) and Night 2 (shown in (**B**)). The relationship between the percentage of stage N1 and stage N2 EEG activation at the O2 electrode on Night 1 (shown in (**C**)) and Night 2 (shown in (**D**)). The *p*-values were corrected for multiple comparisons; * *p* < 0.05.

**Figure 2:**
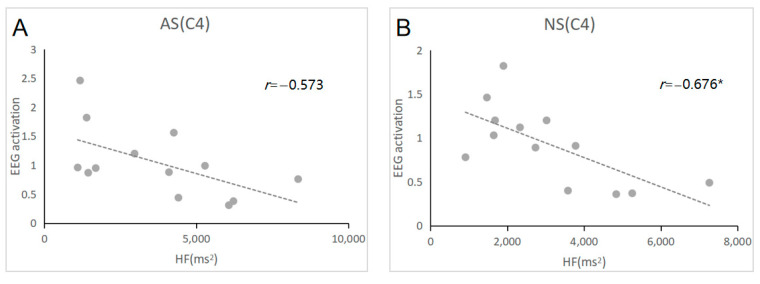
Association between HF and EEG activation during stage N2 at the C4 electrode. Note: Night 1 (shown in (**A**)) and Night 2 (shown in (**B**)). * *p* < 0.05.

We further examined the relationships between EEG activation and heart rate variability and whole-night sleep structure, respectively. For Night 1, there was a significantly positive correlation between the level of EEG activation and the whole-night percentage of stage N1 at the C4 and O2 electrodes (see Figure 1, C4: *r* = 0.667, *p* = 0.04; O2: *r*= 0.577, *p* = 0.05), but these correlations were not significant for Night 2. Since HF-HRV was significantly higher on Night 1 than on Night 2, we calculated the correlation between HF-HRV and EEG activation. The results showed that EEG activation was negatively correlated with HF-HRV at the C4 electrode on Night 2 (see Figure 2; *r* = −0.676, *p* = 0.04) but not on Night 1. At the O2 electrode, there was no significant correlation between HF and EEG activation on Night 1 and Night 2. In addition, we also examined the correlation between EEG activation and WASO, and there was no significant correlation between EEG activation and WASO either on Night 1 or Night 2.

## 4. Discussion

The primary aim of the current study was to examine the FNE during stage N2 and explore the potential mechanism underlying the vulnerability of stage N2 of the FNE. In line with our hypothesis, EEG activation in stage N2 was higher on Night 1 than on Night 2, and the level of EEG activation was positively correlated with the percentage of stage N1 on Night 1. Moreover, we found that HF-HRV increase was caused by the FNE, and the negative correlation between the level of EEG activation and HF-HRV was only shown on Night 2 significantly and not on Night 1. The current findings indicated that increased arousal level and uncoupling of CNS and ANS activity in stage 2 might be associated with the vulnerable stage N2 of the FNE.

### 4.1. The FNE Effect on Increased EEG Activation in Stage N2

The present study revealed that EEG activation was significantly higher during stage N2 on Night 1 than on Night 2. Furthermore, the percentage of stage N1 was also higher on Night 1 than on Night 2 (see Table 2). A series of studies indicated that increased EEG activation is associated with higher cortical arousal levels [16,17,18], as well as a higher percentage of stage N1 [28]. Importantly, we observed the EEG activation was significantly associated with the percentage of stage N1 (see Figure 1). Meanwhile, Shirota and colleagues revealed that the FNE showed higher probabilities of transitioning from stage N2 to stage N1 [7]. Therefore, the present findings might suggest that stage N2 is vulnerable and unstable due to the higher arousal level on the first night, which causes higher probabilities of transitioning from stage N2 to the light sleep stage (stage N1).

Tamaki and colleagues found that the increased lateralization of slow-wave activity in the default-mode network (DMN) is related to a ‘night watch’ system of the FNE, which can assist individuals in waking up from sleep rapidly [5]. In the current study, EEG activation was positively associated with the percentage of stage N1 but not with WASO. Stage N1 is also called light sleep, a state between sleep and wakefulness [22]. Therefore, it is reasonable to speculate that increased EEG activation during stage N2 might be a self-protection mechanism, maintaining the sleep state but keeping a certain arousal level that allows individuals to wake up quickly in unfamiliar sleeping environments.

Furthermore, previous studies have reported that the FNE disrupted EEG activity mainly in the posterior sensory areas and occipital visual areas [4,29]. In line with previous findings, we found that a significant increase in EEG activation was localized in the right central and occipital areas, which may reflect higher arousal levels in the sensorimotor and visual cortexes during stage N2 on Night 1. Moreover, in the current study, the arousal level increased only in the right hemisphere, which may be due to the right hemisphere’s role during sleep. It has been shown that the right hemisphere monitors potential warning stimuli by falling asleep later than the left hemisphere during sleep [30]. The current results further demonstrated that the right sensory and motor cortexes also have a ‘night watch’ role on the FNE, which is reflected in enhanced arousal levels. Individuals could keep alert to external environment information during sleep [31,32]. Thus, we could further propose that the vulnerability and instability of stage N2 of the FNE might be a self-protection mechanism to adapt to new environments by enhancing the arousal level of the right central and occipital cortex.

### 4.2. The FNE Effect of Uncoupling between CNS and ANS Activity in Stage N2

The current results showed that HF-HRV during stage N2 was higher on Night 1 than on Night 2, but no differences in other parameters of HRV were observed between Night 1 and Night 2. HF-HRV is the marker of parasympathetic nervous system activity, and the parasympathetic nervous system is associated with restorative functions and maintains ongoing sleep by reducing autonomic arousal levels [33]. Therefore, increased HF-HRV during stage N2 may reflect the effort to resist the influence of the FNE on increased EEG activation.

In the current study, EEG activation was negatively associated with HF-HRV only during stage N2 of Night 2. This result was in line with the stable coupling effect between CNS and ANS activity during normal sleep [12,13]. However, on Night 1, both the EEG activation index and HF-HRV were increased, and no such significant association was revealed, which might reflect an imbalance state in which the ANS tries to fall asleep and the CNS struggles to maintain arousal due to the FNE. Similarly, previous studies found that the relationship between CNS and ANS activity was decreased in patients with sleep disorders [34,35], which might be related to the higher arousal level of the patients during sleep. Furthermore, the CNS-ANS coupling is closely associated with stable sleep states [10]. Therefore, we proposed that the uncoupling of the CNS and ANS on Night 1, reflected in the decreased relationship between EEG activation and HF-HRV, may also contribute to the vulnerability and instability of stage N2. Our study highlights the importance of the link between the CNS and ANS on the FNE. Since the incidences of the FNE were diverse among clinical patients with different sleep disorders (including insomnia, hypersomnia, obstructive sleep apnea, and restless legs syndrome) [36], the current findings might be helpful to evaluate the FNE in the clinical context more precisely, not only with the indexes of CNS activity, but also with the changes in ANS activity.

### 4.3. Limitations and Future Directions

This research had several limitations. First, the sample size of this study was relatively small, which might weaken the extent of generalization on the basis of the present findings, even though the effect sizes of significant variables were reliable. In addition, because we initially applied the EEG activation index to investigate the FNE, the current study controlled other potential factors, such as limiting age and occupation, to reveal the association between the FNE and EEG activation index and HRV. Although no direct evidence demonstrated the effect of occupation on the FNE, a recent meta-analysis of the FNE suggested that young adults had lower FNE levels than other populations [37]. This might limit the implication scope of the current findings. Future studies should consider the influence of social factors on FNE, such as age, gender, and occupation.

Second, we only used six electrodes to record the whole-night EEG data, such that EEG activation could not be mapped extremely accurately in terms of both spatial and temporal resolution. In a future study, it would be quite interesting to add more electrodes to collect data during sleep, which may provide more information on the FNE; for instance, HD-EEG could be used to localize brain areas that are activated differently [38], and potential changes in EEG activation indexes related to the FNE could be investigated simultaneously.

Finally, the previous study verified individual differences in susceptibility to the FNE [19]. Limited by the relatively small size, it is difficult to examine the individual differences between CNS and ANS activities caused by the FNE with the current sample. Future studies may consider applying larger samples to explore the neurophysiological mechanism of susceptibility to the FNE.

## 5. Conclusions

By observing the activity of the CNS and ANS, this study investigated the vulnerability of stage N2 caused by the FNE. The results showed that the FNE increased both EEG activation and HRV in stage N2. Furthermore, the correlation between EEG activation and HRV in stage N2 was decreased by the FNE. The current findings indicate that the FNE affects the instability of stage N2 by increasing CNS activity and uncoupling the activity between the CNS and ANS, which provides a new perspective to evaluate the FNE in both healthy and clinical populations.

## Figures and Tables

**Table 1:** The average results of the sleep diaries from one week preceding the study.

	Sleeping Time	Waking Time	Sleep Duration (h)
Day 1	24:03 ± 0.25	7:36 ± 0.33	7.35 ± 0.73
Day 2	24:00 ± 0.44	7:42 ± 0.52	7.50 ± 0.76
Day 3	23:59 ± 0.27	7:43 ± 0.35	7.55 ± 0.53
Day 4	24:06 ± 0.25	7:47 ± 0.46	7.42 ± 0.67
Day 5	23:59 ± 0.29	7:35 ± 0.35	7.34 ± 0.43
Day 6	23:55 ± 0.28	7:33 ± 0.25	7.44 ± 0.45
Day 7	24:01 ± 0.30	7:34 ± 0.24	7.38 ± 0.38

Note: Data are presented as means ± SDs.

**Table 2:** Polygraphic sleep structure on Night 1 and Night 2.

	Night 1	Night 2	*t*_(16)_	*d*
Time in bed (min)	487.45 ± 32.75	477.29 ± 17.61	1.351	0.33
Total sleep time (min)	436.88 ± 39.08	457.91 ± 14.88	−2.075	0.50
Sleep efficiency (%)	89.65 ± 5.74	95.98 ± 2.13	−4.980 ***	1.21
Sleep latency (min)	18.26 ± 13.48	8.50 ± 7.45	3.566 **	0.86
REM latency (min)	98.56 ± 56.16	77.53 ± 34.01	1.331	0.32
Stage 1 (%)	7.13 ± 3.14	4.88 ± 2.17	4.393 ***	1.07
Stage 2 (%)	50.42 ± 5.56	52.47 ± 7.06	−1.301	0.32
Stage 3 (%)	19.75 ± 7.85	19.31 ± 5.99	0.250	0.06
REM (%)	22.65 ± 3.71	22.77 ± 3.68	−0.105	0.03
WASO (min)	32.21 ± 20.92	10.85 ± 6.88	4.597 ***	1.11

Note: Data are presented as means ± SDs. Condition differences assessed with paired *t*-tests (two-tailed). WASO, wakefulness after sleep onset; REM, rapid eye movement. ** *p* < 0.01, *** *p* < 0.001.

**Table 3:** The EEG activation index results for stage N2 on Night 1 and Night 2.

	Night 1	Night 2	*t*_(15)_	*d*
F3	0.661 ± 0.35	0.593 ± 0.30	1.472	0.37
F4	0.663 ± 0.35	0.621 ± 0.28	0.876	0.22
C3	1.035 ± 0.65	0.937 ± 0.49	1.303	0.33
C4	1.027 ± 0.55	0.891 ± 0.41	2.322 *	0.61
O1	1.178 ± 0.71	1.168 ± 0.57	0.097	0.03
O2	1.235 ± 0.75	1.049 ± 0.52	2.337 *	0.59

Note: Data are presented as means ± SDs. Condition differences assessed with paired *t*-tests (two-tailed). * *p* < 0.05.

**Table 4:** The HRV results for stage N2 on Night 1 and Night 2.

	Night 1	Night 2	*t*_(13)_	*d*
RR intervals	1145.5 ± 120.14	1139.07 ± 112.11	0.567	0.15
HR	52.79 ± 5.32	53.21 ± 4.93	−0.822	0.22
LF	1977.86 ± 1226.16	1810.64 ± 1024.99	1.348	0.36
HF	3823.36 ± 2268.45	3242.64 ± 1814.43	3.100 **	0.83
LF/HF	0.76 ± 0.69	0.69 ± 0.49	1.135	0.31

Note: Data are presented as means ± SDs. Condition differences assessed with paired *t*-tests (two-tailed). HR (ms), heart rate; LF, low frequency (ms^2^); HF, high frequency (ms^2^); LF/HF, low frequency/high frequency. ** *p* < 0.01.

## Data Availability

The datasets of the current study are available from the corresponding authors on reasonable request.
